# Work-family life course trajectories and mental well-being: evidence from CHARLS, SHARE, ELSA, HRS, NCDS and BCS70

**DOI:** 10.1093/geroni/igaf122.4407

**Published:** 2025-12-31

**Authors:** Jiajia Li, Fabrice Kämpfen, Heming Pei

**Affiliations:** Institute of Population Research, Peking University, Beijing, Beijing, China (People’s Republic); School of Economics, University College Dublin, Dublin, Hofuoborgarsvaoio, Iceland; UNC Gillings School of Global Public Health University of North Carolina at Chapel Hill, Chapel Hill, North Carolina, United States

## Abstract

Later-life mental well-being is shaped by cumulative work, partnership, and parenting histories, yet few studies compare these life-course patterns across diverse regions using harmonized designs. We analyzed 61,132 adults aged ≥50 from four HRS-sister studies—the China Health and Retirement Longitudinal Study (CHARLS;n=9,276), Survey of Health, Ageing and Retirement in Europe (SHARE;n=35,087), English Longitudinal Study of Ageing (ELSA;n=5,283), and Health and Retirement Study (HRS;n=11,486)—and supplemented with two prospective British birth cohorts for robustness. Using multi-channel sequence analysis and hidden Markov mixture models, we identified 5–6 work–family trajectory clusters from ages 15 to 50. Stable full-time employment, sustained partnerships, and one to two children were consistently linked to better outcomes. In CHARLS, the agricultural, early marriage, ≥5 children cluster had depressive symptoms 0.271 SD higher (95% CI:0.233–0.310) and self-rated health 0.108 SD lower (95%CI:−0.138 to − 0.077) than the stable non-agricultural, later marriage,1–2 children group. In SHARE, the Home/family+Early Stable+2-Kids cluster had depressive symptoms 0.076 SD higher (95%CI:0.059–0.094) and self-rated health 0.045 SD lower (95% CI:−0.061to −0.029) than the full-time, early stable, two-child cluster. In HRS, the Home/family focus+Early Diverse + ≥3 children cluster had depressive symptoms 0.227 SD higher (95%CI:0.188–0.267) and self-rated health 0.219 SD lower (95%CI:−0.257 to − 0.181) than the stable reference. Gender disparities were largest in China and smallest in the U.S. Results were robust to early-life adjustment, attrition weighting, and exclusion of adult mediators. This cross-national design highlights the mental-health benefits of stable, balanced work–family patterns and supports policies promoting employment stability, gender equity, and caregiving support across the life course.

